# The Adulteration of Sulphate of Quinine

**Published:** 1840-07

**Authors:** 


*On the Adulteration of the Sulphate of Quinine.*—The recent rise in the price of sulphate of quinine has induced many unprincipled venders of drugs to adulterate it with various ingredients; to such an extent, indeed, has this been carried, in some instances, that not more than a fifth part of what was sold as sulphate of quinine really consisted of that substance. M. Vallet found that the substance chiefly used for the adulteration of the sulphate of quinine was Mannite, a substance similar in external appearance to the sulphate of quinine, but destitute of all the valuable properties for which quinine is so justly celebrated. He found, however, that the adulteration could be with great ease detected by means of pure alcohol, which dissolves the quinine alone, but leaves untouched the mannite, which, however, is freely soluble in water and of its characteristic sweet taste.

M. Dubail has also arrived at the same conclusions as M. Vallet, and in pursuing his investigations, met with one sample of the sulphate of quinine, which, though presenting all the external characters of the genuine unadulterated drug, both as regarded its lightness and silky appearance, did not contain above a sixth part of its weight of sulphate of quinine. The rest was composed of mannite, insoluble in alcohol, but soluble in water, and of a sweet taste.

M. Pelletier has found that that which is sold in sealed packages, with the impress of his own seal, has also been subjected to adulteration, but the substance used in this instance, is gypsum. The same test, however, viz. the solubility in alcohol, applies to this case, so that every druggist would do well to apply this simple test to the sample of this drug before purchasing.—*Edinburgh Med. and Surg. Jour., from Journal de Pharmacie*, January, 1840.

